# Associations between Peritonsillar Abscess and Deep Neck Infection in Chronic Periodontitis Patients: Two Nested Case—Control Studies Using a National Health Screening Cohort

**DOI:** 10.3390/jcm13082166

**Published:** 2024-04-09

**Authors:** So Young Kim, Il Hwan Park, Chun Sung Byun, Hyo Geun Choi, Mi Jung Kwon, Ji Hee Kim, Joo-Hee Kim, Chang Wan Kim

**Affiliations:** 1 Department of Anatomy and Cell Biology, Seoul National University College of Medicine, Seoul 03080, Republic of Korea; sossi81@snu.ac.kr; 2 Department of Cardiovascular and Thoracic Surgery, Yonsei University Wonju College of Medicine, Wonju 26426, Republic of Korea; nicecs@yonsei.ac.kr (I.H.P.); csbyun@yonsei.ac.kr (C.S.B.); 3 Mdanalytics, Seoul 06349, Republic of Korea; pupen@naver.com; 4 Suseoseoulent Clinic, Seoul 06349, Republic of Korea; 5 Department of Pathology, Hallym University Sacred Heart Hospital, Hallym University College of Medicine, Anyang 14068, Republic of Korea; mulank99@hallym.or.kr; 6 Department of Neurosurgery, Hallym University Sacred Heart Hospital, Hallym University College of Medicine, Anyang 14068, Republic of Korea; kimjihee@hallym.or.kr; 7 Department of Medicine, Hallym University Sacred Heart Hospital, Hallym University College of Medicine, Anyang 14068, Republic of Korea; luxjhee@hallym.or.kr

**Keywords:** deep neck infection, peritonsillar abscess, chronic periodontitis, risk factors, case–control studies, epidemiology

## Abstract

**Background/Introduction:** Odontogenic infection is one of the main etiologies of deep neck infection (DNI). However, the relationship between chronic periodontitis (CP) and the incidence of DNI has not been examined. This study aimed to evaluate the incidence of DNI and peritonsillar abscess (PTA) after CP. **Methods:** The Korean National Health Insurance Service-National Sample Cohort 2002–2019 was used. In Study I, 4585 PTA patients were matched with 19,340 control I participants. A previous history of CP for 1 year was collected, and the odds ratios (ORs) of CP for PTA were analyzed using conditional logistic regression. In Study II, 46,293 DNI patients and 185,172 control II participants were matched. A previous history of CP for 1 year was collected, and conditional logistic regression was conducted for the ORs of CP for DNI. Secondary analyses were conducted in demographic, socioeconomic, and comorbidity subgroups. **Results:** In Study I, a history of CP was not related to the incidence of PTA (adjusted OR = 1.28, 95% confidence interval [CI] = 0.91–1.81). In Study II, the incidence of DNI was greater in participants with a history of CP (adjusted OR = 1.55, 95% CI = 1.41–1.71). The relationship between CP history and DNI was greater in groups with young, male, low-income, and rural residents. **Conclusions**: A prior history of CP was associated with a high incidence of DNI in the general population of Korea. Patients with CP need to be managed for the potential risk of DNI.

## 1. Introduction

Deep neck infection (DNI) is an infection of deep neck spaces that can result in several complications and airway problems [1]. DNI often occurs in low-resistance potential neck spaces between fascial layers; thus, it easily spreads to adjacent deep neck spaces, which can result in upper airway obstruction and sepsis [2]. Because of potential complications and life-threatening conditions, prompt incision and drainage of abscesses with microbial culture can be used to identify etiologic pathogens [3]. The peak age of incidence of DNI was estimated to be in the middle-aged population, with a male predominance [3]. Some of the most common sources of DNI are odontogenic, tonsillar, salivary gland, and foreign body sources, as well as malignant sources [1,4]. Odontogenic infection accounts for approximately 42% of the etiologic factors of DNI and is related to poor oral hygiene, chronic irritation by dental caries, and tobacco smoking [3].

Peritonsillar abscess (PTA) is the most common DNI and refers to abscess formation between the tonsillar capsule and the pharyngeal constrictor muscle [5]. PTA may originate from acute tonsillitis and/or the obstruction of the common duct from Weber’s glands [5]. Middle age, a history of recurrent tonsillitis, and insufficient antibiotic treatment were reported as risk factors for recurrent PTA [6]. In addition, tobacco smoking was suggested to be a risk factor for PTA formation [7]. Although most PTAs can be cured by drainage of the abscess and appropriate antibiotic treatment, PTA can be spread to other deep neck spaces with complications, such as necrotizing mediastinitis [8].

Chronic periodontitis (CP) is a chronic inflammatory disease of the periodontium, including the gingiva, cementum, periodontal ligament, and alveolar bone [9]. According to the recent disease classification system, CP is considered on a spectrum of clinical periodontal health at various site levels [10]. Low-grade inflammation can be developed as a multifactorial chronic inflammatory disease according to the immune and inflammatory response of the individuals [11]. Due to chronic inflammatory conditions characterized by the secretion of cytokines such as tumor necrosis factor-alpha, interleukin-1, and interleukin-6, CP may be interrelated with other chronic inflammatory diseases, such as chronic kidney disease and multiple sclerosis [9,12]. In addition, because CP involves inflammation in the oral cavity, we can suppose that there is an increased incidence of oral cavity infections, such as PTA and DNI.

Several previous studies have reported on ontogenic infections associated with PTA and DNI [13,14,15]. However, no study has investigated the incidence of PTA or DNI after CP. To fill this knowledge gap, this study investigated the incidence of PTA and DNI after CP in a large population cohort. This study randomly selected and matched the control group with the study population to avoid selection bias. In addition, secondary analyses were conducted to identify the differential associations of CP with PTA and DNI according to demographic variables, socioeconomic factors, and comorbidities.

## 2. Methods

### 2.1. Ethical Consideration

The ethics committee of Hallym University (2022-10-008, approval date: 8 October 2022) approved the analysis of the current data. The requirement for written informed consent was waived by the Institutional Review Board. The analyses were conducted in accordance with the rules of the ethics committee of Hallym University.

### 2.2. Chronic Periodontitis (Outcome)

CP was included using the ICD-10 code K05.3, which was determined by a physician [16]. The treatment history of CP was collected for 1 year before the diagnosis of PTA or DNI.

### 2.3. Peritonsillar Abscess (Exposure)

PTA was included based on the ICD-10 code (J36) determined by a physician. In addition, patients with PTA were defined based on the history of incision and drainage or aspiration for the PTA (claim code: Q2320) [17].

### 2.4. Deep neck Infection (Exposure)

The DNI included retropharyngeal or parapharyngeal abscess, which was diagnosed with the ICD-10 codes J39.0 and J39.1 by a physician [18].

### 2.5. Study Population

This study analyzed the Korean National Health Insurance Service-National Sample Cohort (NHIS-NSC) from 2002 to 2019, which included demographic and socioeconomic data, diagnostic codes, and histories of procedures [19].

#### 2.5.1. Study I

**Figure 1:**
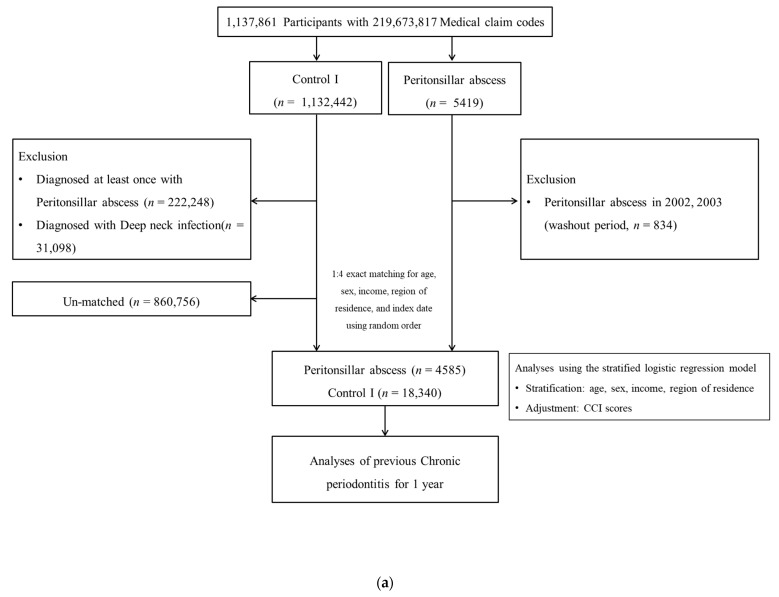

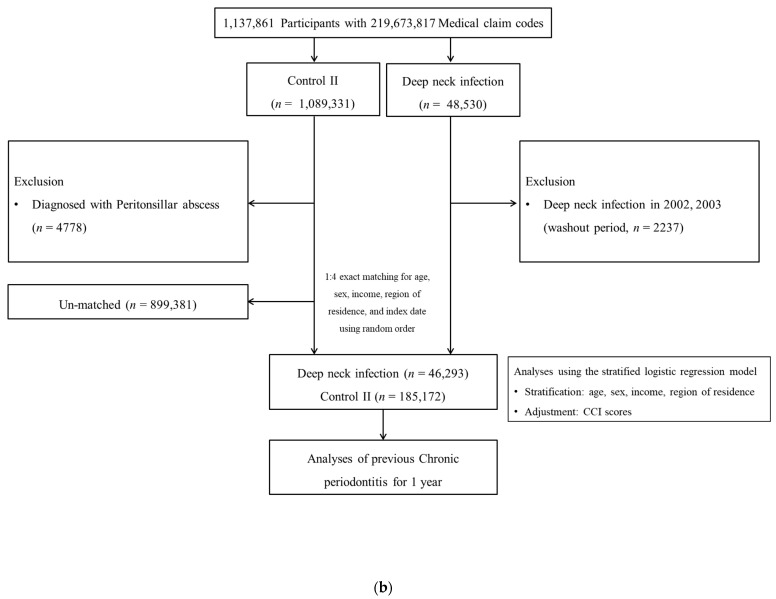
(**a**) A schematic illustration of the participant selection process that was used in the present study. Of a total of 1,137,861 participants, 4585 participants with peritonsillar abscess were matched with 18,340 control participants for age, sex, income, and region of residence. (**b**) A schematic illustration of the participant selection process that was used in the present study. Of a total of 1137,861 participants, 46,293 participants with deep neck infection were matched with 185,172 control participants for age, sex, income, and region of residence.

A total of 5419 PTA participants were recruited from the NHIS-NSC (2002–2019). A total of 11,32,442 participants who did not satisfy the diagnostic criteria for PTA were enrolled. In the PTA group, 834 PTA patients who had a history of PTA between 2002 and 2003 were removed. Among the control participants, 222,248 with diagnostic codes for PTA and 31,098 with a history of DNI were removed. Matching procedures were conducted for demographic and socioeconomic variables between the PTA group and the control I group. A total of 860,756 control I participants were removed after matching with the PTA group. A total of 4585 and 18,340 participants were selected for the PTA and control I groups, respectively (Figure 1a).

#### 2.5.2. Study II

A total of 48,530 DNI participants were included. A total of 1,132,442 participants who did not meet the criteria for DNI were selected. For the DNI group, the 2237 DNI patients who were diagnosed between 2002 and 2003 were removed. A total of 4778 participants who had a history of PTA were removed. Matching procedures were conducted for demographic and socioeconomic variables. A total of 899,381 control participants were removed after the matching procedure. The 46,293 and 185,172 participants were defined as the DNI and control II groups, respectively (Figure 1b).

### 2.6. Variables

The 18 age groups were allocated at 5-year intervals. Based on the health insurance system categorization, the level of income was divided into 5 groups. Urban and rural residents were categorized according to the registered data in the health insurance system [20].

To evaluate the comorbidities, 17 types of comorbidities were counted using the Charlson comorbidity index (CCI) score and analyzed as continuous variables [21].

### 2.7. Statistical Methods

The standardized difference (SD) was calculated to estimate the difference in the variables between the study and control groups.

Conditional logistic regression analysis was performed to estimate odds ratios (ORs) with 95% confidence intervals (CIs) of CP for PTA and DNI. The CCI scores were adjusted, and age, sex, income, and region of residence were stratified.

Secondary analyses were conducted in accordance with all of the variables included.

All analyses were two-tailed, and a *p*-value < 0.05 was considered to indicate statistical significance. All analyses were conducted using SAS version 9.4 (SAS Institute Inc., Cary, NC, USA).

## 3. Results

### 3.1. Study I

The average numbers of CP treatments were 0.26 (SD = 0.90) in the PTA group and 0.24 (SD = 0.92) in the control group (SD = 0.02, Table 1). The mean CCI scores were 0.37 (SD = 1.03) and 0.31 (SD = 0.98) in the PTA and control groups, respectively (SD = 0.05). Other variables were matched between the PTA and control groups (SD = 0.00).

The number of CP treatments was not associated with high odds for PTA (adjusted OR = 1.28, 95% CI = 0.91–1.81, *p* = 0.154; Table 2). According to the subgroup analyses, only the rural residence group demonstrated greater odds for PTA related to the number of CP treatments (adjusted OR = 1.84, 95% CI = 1.13–2.97; *p* = 0.013). None of the other subgroups showed a relationship between the number of CP treatments and the odds of PTA (all *p* > 0.05).

### 3.2. Study II

The average number of CP treatments was greater in the DNI group than in the control group (0.33 [SD = 1.05] in the DNI group and 0.28 [0.98] in the control group, SD = 0.04; Table 3). The CCI score was greater in the DNI group than in the control group (0.33 [SD = 0.96] in the DNI group and 0.31 [SD = 0.96] in the control group, SD = 0.02).

The odds for DNI were greater for participants with more CP treatments (adjusted OR = 1.55, 95% CI = 1.41–1.71, *p* < 0.001; Table 4). The higher odds for DNI related to CP were consistent in all subgroups of age, sex, income, residence, and CCI scores. According to age, the younger age group (<35 years old) had greater odds for DNI among participants with a history of CP (adjusted OR [95% CI] = 1.76 [1.36–2.29] in the <35 years old group vs. 1.52 [1.36–1.69] in the ≥35 years old group). The male group had greater odds of having a history of DNI related to CP (adjusted OR [95% CI] =1.63 [1.41–1.88] in the male group vs. 1.48 [1.30–1.70] in the female group). The lower-income group presented greater odds for DNI related to CP (adjusted OR [95% CI] = 1.64 [1.42–1.90] in the low-income group vs. 1.48 [1.29–1.69] in the high-income group). The rural residence group had greater odds of having DNI related to CP (adjusted OR [95% CI] = 1.65 [1.44–1.89] > 1.45 [1.25–1.67]).

## 4. Discussion

A prior history of CP was associated with an increased risk of DNI in the Korean population. However, there was no relationship between a history of CP and PTA, except among rural residents. To our knowledge, no previous study has investigated the potential risk of DNI or PTA related to CP history. This study used large, nationwide population data sets, which permitted matching of the control participants and minimized selection bias during the selection of the control group. In addition, secondary analyses were conducted, and age, sex, and socioeconomic characteristics that showed differential impacts on the relationship between DNI and CP were elucidated in the present study.

The presence of CP influenced the subsequent risk of DNI in the present study. Although no study has reported on the risk of DNI in patients with CP, there has been growing evidence on the impacts of odontogenic infections on DNI [22,23]. The submandibular space is the most common deep neck space affected by odontogenesis [22]. The anatomical proximity may impose a relationship between CP and DNI. Moreover, chronic inflammatory conditions in CP may increase the risk of new infection in the deep neck space. In CP patients, subgingival dental biofilms induce inflammation and an immune response, which irreversibly destroys the periodontium [22]. In addition to local inflammation, inflammatory factors and cytokine pathways have been identified to lead to systemic inflammation [24]. For instance, the C-X3-C motif ligand 1/chemokine receptor 1 axis accelerates the occurrence of periodontitis, and the levels of these chemokines are elevated in multiple samples, such as gingival tissue, saliva, and serum [24]. C-X3-C motif ligand 1/chemokine receptor 1 axis plays a role as a cell adhesion molecule and a chemoattractant for immune cells [24]. Last, common risk factors, such as poor hygiene or an immune-compromised state, can mediate the link between CP and DNI.

The demographic and socioeconomic features of younger age, male sex, low income, and rural residence were associated with greater odds of DNI following CP history in this study. The characteristics of DNI differ according to age group. It has been reported that the elderly population, who require surgical treatment and have a long duration of hospital stays, have greater incidences of multiple types of space involvement, complications, and severe complications than younger people [25,26]. Moreover, the microbiology of DNI differs between adults and children [27]. Compared to children, adults harbor more polymicrobial infections in their abscess drainage of DNI [27]. Thus, it can be supposed that in the old age group, more complex factors can evoke DNI of other than odontogenic origin than in the young age group. According to sex, the incidence of DNI was greater in males [27]. This high incidence of DNI can impact the strong relationship between CP and DNI by enhancing the statistical power. In the socioeconomic environment, low socioeconomic status is supposed to increase susceptibility to DNI [28]. This high vulnerability to DNI can influence the current results on the relationship between CP and DNI.

This study is based on NHIS-NSC data, which are composed of national health claim codes. All Koreans are obligatorily registered in the NHIS, and these data are the reference data for reimbursement of medical costs in Korea. Thus, there was little concern about missing data in the current study. The NHIS-NSC data were selected by statisticians in the Korean government and validated for representative selection of participants. However, because NHIS data were collected from participants who visited clinics, participants who did not visit clinics were excluded from the present study. The diagnoses of CP and DNI were based on the diagnostic codes determined by a physician. Thus, these methods are objective but can include various disease severities and subtypes. We could not classify periodontitis as provided by the American Academy of Periodontology and the European Federation of Periodontology [10]. We could not include the treatment histories of medication and surgical procedures for CP and DNI, which may have led to heterogeneity in the analyses. Although we included several variables, including demographic and socioeconomic factors and comorbidities, there are several potential confounders, such as a history of tonsillectomy or other neck surgeries and a history of airway infection, that can influence the relationship between CP and DNI [29,30]. Further studies may be warranted to determine the specific associations between CP and DNI according to severity and subtype. Finally, although we retrospectively followed up on the history of CP before the diagnosis of DNI, we could not determine the causal relationship between CP and DNI. A randomized controlled study with a prospective design needs to be conducted to address the current limitations.

## 5. Conclusions

A prior history of CP was linked to a high incidence of DNI in the Korean population. Young age, male sex, low income level, and rural residence were strongly associated with CP and subsequent DNI. In the clinic, patients with CP need to be managed with consideration of the risk of DNI.

## Figures and Tables

**Table 1:** General characteristics of participants.

Characteristics	Total Participants		
	PTA (*n*, %)	Control I (*n*, %)	Standardized Difference
Total number	4585 (100.0)	18,340 (100.0)	
Age (*n*, %)			0.00
0–4	12 (0.26)	48 (0.26)	
5–9	59 (1.29)	236 (1.29)	
10–14	175 (3.82)	700 (3.82)	
15–19	466 (10.16)	1864 (10.16)	
20–24	488 (10.64)	1952 (10.64)	
25–29	618 (13.48)	2472 (13.48)	
30–34	543 (11.84)	2172 (11.84)	
35–39	510 (11.12)	2040 (11.12)	
40–44	453 (9.88)	1812 (9.88)	
45–49	331 (7.22)	1324 (7.22)	
50–54	279 (6.09)	1116 (6.09)	
55–59	215 (4.69)	860 (4.69)	
60–64	166 (3.62)	664 (3.62)	
65–69	126 (2.75)	504 (2.75)	
70–74	71 (1.55)	284 (1.55)	
75–79	48 (1.05)	192 (1.05)	
80–84	18 (0.39)	72 (0.39)	
85+	7 (0.15)	28 (0.15)	
Sex (*n*, %)			0.00
Male	2750 (59.98)	11,000 (59.98)	
Female	1835 (40.02)	7340 (40.02)	
Income (*n*, %)			0.00
1 (lowest)	844 (18.41)	3376 (18.41)	
2	770 (16.79)	3080 (16.79)	
3	886 (19.32)	3544 (19.32)	
4	994 (21.68)	3976 (21.68)	
5 (highest)	1091 (23.79)	4364 (23.79)	
Region of residence (*n*, %)			0.00
Urban	2179 (47.52)	8716 (47.52)	
Rural	2406 (52.48)	9624 (52.48)	
CCI score (Mean, SD)	0.37 (1.03)	0.31 (0.98)	0.05
Number of CP treatments (Mean, SD)	0.26 (0.90)	0.24 (0.92)	0.02

Abbreviations: CCI, Charlson comorbidity index; CP, chronic periodontitis.

**Table 2:** Crude and adjusted odds ratios for PTA in the number of treatments for CP (per 10 treatments) with subgroup analyses according to age, sex, income, region of residence, and CCI scores.

Characteristics	Odds Ratios for PTA (95% Confidence Interval)			
	Crude †	*p*-Value	Adjusted ‡	*p*-Value
Total participants (*n* = 22,925)	1.27 (0.90–1.79)	0.175	1.28 (0.91–1.81)	0.154
Age < 35 years old (*n* = 11,805)	1.43 (0.61–3.35)	0.415	1.45 (0.62–3.41)	0.392
Age ≥ 35 years old (*n* = 11,120)	1.24 (0.85–1.81)	0.261	1.25 (0.86–1.83)	0.241
Male (*n* = 13,750)	1.28 (0.84–1.95)	0.243	1.29 (0.85–1.95)	0.238
Female (*n* = 9175)	1.24 (0.68–2.29)	0.485	1.30 (0.70–2.39)	0.404
Low-income group (*n* = 12,500)	1.30 (0.78–2.16)	0.307	1.31 (0.79–2.18)	0.297
High-income group (*n* = 10,425)	1.24 (0.78–1.99)	0.366	1.27 (0.79–2.03)	0.328
Urban residents (*n* = 10,895)	0.89 (0.54–1.47)	0.651	0.91 (0.55–1.51)	0.716
Rural residents (*n* = 12,030)	1.83 (1.13–2.96)	0.014 *	1.84 (1.13–2.97)	0.013 *
CCI scores = 0 (*n* = 19,224)	1.17 (0.79–1.73)	0.444	1.20 (0.80–1.79)	0.384
CCI scores = 1 (*n* = 2071)	1.01 (0.41–2.51)	0.986	1.33 (0.53–3.32)	0.547
CCI scores ≥ 2 (*n* = 1630)	2.02 (0.75–5.40)	0.163	2.22 (0.82–6.01)	0.117

Abbreviations: CCI, Charlson comorbidity index; * conditional or unconditional logistic regression model, significance at *p* < 0.05. † Models were stratified by age, sex, income, and region of residence. ‡ Adjusted for CCI scores.

**Table 3:** General characteristics of participants.

Characteristics	Total Participants		
	DNI (*n*, %)	Control II (*n*, %)	Standardized Difference
Total number	46,293 (100.0)	185,172 (100.0)	
Age (*n*, %)			0.00
0–4	3265 (7.05)	13,060 (7.05)	
5–9	2853 (6.16)	11,412 (6.16)	
10–14	2616 (5.65)	10,464 (5.65)	
15–19	3189 (6.89)	12,756 (6.89)	
20–24	3023 (6.53)	12,092 (6.53)	
25–29	3922 (8.47)	15,688 (8.47)	
30–34	4657 (10.06)	18,628 (10.06)	
35–39	4727 (10.21)	18,908 (10.21)	
40–44	3903 (8.43)	15,612 (8.43)	
45–49	3303 (7.13)	13,212 (7.13)	
50–54	2973 (6.42)	11,892 (6.42)	
55–59	2483 (5.36)	9932 (5.36)	
60–64	1931 (4.17)	7724 (4.17)	
65–69	1373 (2.97)	5492 (2.97)	
70–74	982 (2.12)	3928 (2.12)	
75–79	637 (1.38)	2548 (1.38)	
80–84	323 (0.70)	1292 (0.70)	
85+	133 (0.29)	532 (0.29)	
Sex (*n*, %)			0.00
Male	20,777 (44.88)	83,108 (44.88)	
Female	25,516 (55.12)	102,064 (55.12)	
Income (*n*, %)			0.00
1 (lowest)	7331 (15.84)	29,324 (15.84)	
2	6621 (14.30)	26,484 (14.30)	
3	8808 (19.03)	35,232 (19.03)	
4	11,028 (23.82)	44,112 (23.82)	
5 (highest)	12,505 (27.01)	50,020 (27.01)	
Region of residence (*n*, %)			0.00
Urban	21,228 (45.86)	84,912 (45.86)	
Rural	25,065 (54.14)	100,260 (54.14)	
CCI score (Mean, SD)	0.33 (0.96)	0.31 (0.96)	0.02
Number of CP treatments (Mean, SD)	0.33 (1.05)	0.28 (0.98)	0.04

Abbreviations: CCI, Charlson comorbidity index.

**Table 4:** Crude and adjusted odds ratios for DNI in the number of treatments for CP (per 10 treatments) with subgroup analyses according to age, sex, income, region of residence, and CCI scores.

Characteristics	Odds Ratios for DNI (95% Confidence Interval)			
	Crude †	*p*-Value	Adjusted ‡	*p*-Value
Total participants (*n* = 231,465)	1.54 (1.40–1.70)	<0.001 *	1.55 (1.41–1.71)	<0.001 *
Age < 35 years old (*n* = 117,625)	1.76 (1.36–2.29)	<0.001 *	1.76 (1.36–2.29)	<0.001 *
Age ≥ 35 years old (*n* = 113,840)	1.51 (1.36–1.68)	<0.001 *	1.52 (1.36–1.69)	<0.001 *
Male (*n* = 103,885)	1.62 (1.40–1.87)	<0.001 *	1.63 (1.41–1.88)	<0.001 *
Female (*n* = 127,580)	1.48 (1.29–1.69)	<0.001 *	1.48 (1.30–1.70)	<0.001 *
Low-income group (*n* = 113,800)	1.64 (1.41–1.89)	<0.001 *	1.64 (1.42–1.90)	<0.001 *
High-income group (*n* = 117,665)	1.47 (1.29–1.68)	<0.001 *	1.48 (1.29–1.69)	<0.001 *
Urban residents (*n* = 106,140)	1.44 (1.25–1.67)	<0.001 *	1.45 (1.25–1.67)	<0.001 *
Rural residents (*n* = 125,325)	1.64 (1.43–1.88)	<0.001 *	1.65 (1.44–1.89)	<0.001 *
CCI scores = 0 (*n* = 194,230)	1.52 (1.36–1.70)	<0.001 *	1.56 (1.40–1.75)	<0.001 *
CCI scores = 1 (*n* = 21,585)	1.19 (0.89–1.58)	0.239	1.35 (1.01–1.81)	0.043 *
CCI scores ≥ 2 (*n* = 15,650)	1.57 (1.20–2.06)	0.001 *	1.61 (1.23–2.12)	<0.001 *

Abbreviations: CCI, Charlson comorbidity index; * conditional or unconditional logistic regression model, significance at *p* < 0.05. † Models were stratified by age, sex, income, and region of residence. ‡ Adjusted for CCI scores.

## Data Availability

The datasets presented in this article are not readily available because the data was derived from Korean Health Insurance system. Requests to access the datasets should be directed to NHIS-NSC.
